# Metabolic Control of Treg Cell Stability, Plasticity, and Tissue-Specific Heterogeneity

**DOI:** 10.3389/fimmu.2019.02716

**Published:** 2019-12-11

**Authors:** Hao Shi, Hongbo Chi

**Affiliations:** Department of Immunology, St. Jude Children's Research Hospital, Memphis, TN, United States

**Keywords:** metabolism, Treg cell, Foxp3, stability, plasticity, tissue-specific heterogeneity

## Abstract

Regulatory T (Treg) cells are crucial for peripheral immune tolerance and prevention of autoimmunity and tissue damage. Treg cells are inherently defined by the expression of the transcription factor Foxp3, which enforces lineage development and immune suppressive function of these cells. Under various conditions as observed in autoimmunity, cancer and non-lymphoid tissues, a proportion of Treg cells respond to specific environmental signals and display altered stability, plasticity and tissue-specific heterogeneity, which further shape their context-dependent suppressive functions. Recent studies have revealed that metabolic programs play pivotal roles in controlling these processes in Treg cells, thereby considerably expanding our understanding of Treg cell biology. Here we summarize these recent advances that highlight how cell-extrinsic factors, such as nutrients, vitamins and metabolites, and cell-intrinsic metabolic programs, orchestrate Treg cell stability, plasticity, and tissue-specific heterogeneity. Understanding metabolic regulation of Treg cells should provide new insight into immune homeostasis and disease, with important therapeutic implications for autoimmunity, cancer, and other immune-mediated disorders.

## Introduction

Regulatory T (Treg) cells are critical for the establishment of peripheral tolerance, with altered Treg cell function leading to autoimmune disease and immunopathology (1, 2). Treg cells constitutively express CD25, the α subunit of IL-2 receptor, and require continuous IL-2 signals for homeostasis and function (1–5). The transcription factor forkhead box P3 (Foxp3) is essential for specifying the lineage and suppressive function of Treg cells (1, 2). The majority of peripheral Treg cells originate from the thymus and are known as thymus-derived Treg (tTreg) cells (6–8). Treg cells may also differentiate from naïve CD4^+^ T cells in the periphery [called peripherally-derived Treg (pTreg) cells] or *in vitro* after stimulation in the presence of TGF-β and IL-2 (termed iTreg cells) (6, 9, 10), which are distinguished from tTreg cells by the lack of Helios and neuropilin-1 expression (11–13). In addition, epigenetic modifications of the *Foxp3* locus differ between tTreg and pTreg cells (6, 10). How these Treg cells arise and contribute to Treg cell suppressive function in different contexts has remained an important question for the field.

Recent advances have highlighted the important role of metabolism in immune cells, including Treg cells (14, 15). Initial studies showed that iTreg cells and conventional effector T helper cells (Th1, Th2, and Th17) require fatty-acid oxidation (FAO) and glycolysis, respectively, for their proliferation, differentiation, and survival (16). More recent analysis has shown that Foxp3 expression likely contributes to these effects (17–19). However, Treg cells *in vivo* are more metabolically active than conventional naïve T cells and undergo increased levels of proliferation balanced by apoptosis (20–22). Also, dietary nutrients and metabolites serve as important environmental factors that influence Treg cell function (23). Intracellular metabolites and metabolic pathways also modulate the expression of Foxp3, as well as Treg cell transcriptional programs and functional plasticity (20, 21, 23). In particular, nutrient-fueled mTORC1 activation promotes metabolic reprogramming in Treg cells *in vivo*, with increased lipogenesis and mevalonate pathway-dependent cholesterol biosynthesis to support Treg cell proliferation and function (22, 24). However, inappropriate mTORC1 activation and unconstrained glycolysis in Treg cells lead to decreased Foxp3 expression and reduced Treg cell suppressive activity, indicating that cellular metabolism plays essential roles for regulating Foxp3 stability and Treg cell function (18, 25–27). In this review, we summarize the recent advances that have defined how environmental metabolites and nutrients, as well as cell-intrinsic metabolic programs, orchestrate Treg cell function by affecting stability, plasticity, and tissue-specific heterogeneity.

## Metabolic Regulation of Treg Cell Lineage Stability

Dysfunctional mutations in the *Foxp3* gene result in fatal autoimmunity with Scurfy phenotype in mice and IPEX (Immuno-dysregulation, Polyendocrinopathy, Enteropathy, X-linked) syndrome in humans due to altered Treg cell development (28, 29). However, maintaining Foxp3 expression is also essential for Treg cell function. The majority of Treg cells are a stable population under steady state or upon transfer into environments that contain T cells (30, 31). More recently, the concept of Treg cell stability, which is defined as the ability to maintain Foxp3 expression and resist acquiring pro-inflammatory effector functions during inflammation, has emerged as a crucial determinant of Treg cell function in selective contexts (32–34). For example, Treg cells display considerable loss of stability *in vitro* when stimulated with proinflammatory cytokines, including IL-6 and IL-4 (35, 36). The resultant Foxp3^−^ cells are referred to as “exTreg” cells (35), which are also observed in autoimmune mouse models (37). Adoptive transfer of purified Foxp3^+^ Treg cells into lymphopenic recipients that lack conventional T cells also results in a dramatic loss of Foxp3 expression (30, 37, 38). These Foxp3^−^ cells acquire the expression of inflammatory cytokines and fail to mediate immune suppression (30, 37, 38). Interestingly, the unstable Treg cells are mostly limited to CD25^lo^Foxp3^+^ subset, raising the possibility that a small portion of Treg cells are inherently prone to becoming unstable *in vivo* (30). Further research using fate-mapping mouse models has shown that some exTreg cells are from activated T cells that have transiently expressed Foxp3 and failed to fully differentiate into Treg cells (39), thus establishing stability as a context-dependent regulator of inflammation and peripheral tolerance.

**Figure 1:**
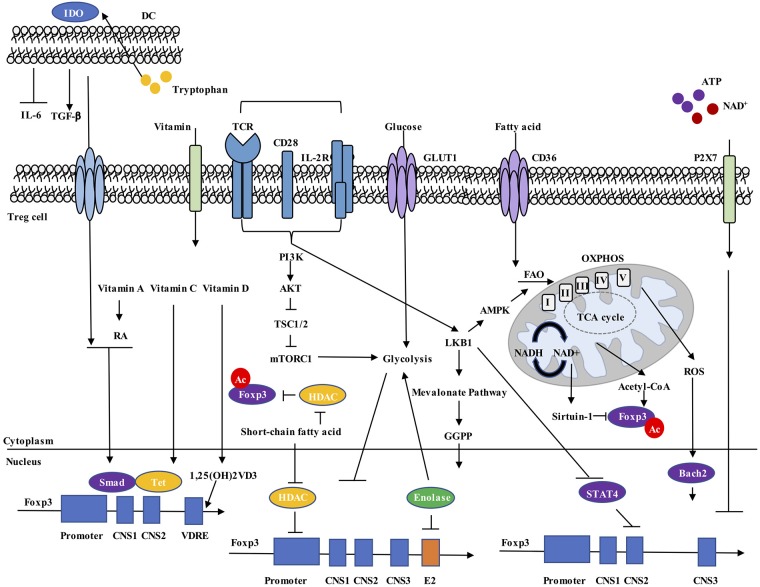
Metabolic regulation of Foxp3 expression. Environmental metabolites, intracellular metabolic intermediates, and signaling pathways all regulate Foxp3 expression in Treg cells. (1) Dendritic cells (DCs) express IDO, drive tryptophan metabolism to promote TGF-β and inhibit IL-6 production, and increase Foxp3^+^ Treg cell generation. (2) Vitamin A metabolite RA, together with TGF-β-induced Smad activation, increase *Foxp3* expression. Vitamin C stabilizes Foxp3 expression through maintaining demethylated state of *Foxp3* CNS2 region by Tet methylcytosine dioxygenase. Vitamin D3 metabolite 1,25(OH)_2_VD_3_ increases *Foxp3* gene expression by binding to VDRE region. (3) Extracellular ATP and NAD^+^ released by cell lysis activate the P2X7 receptor and induce Treg cell instability. (4) Increased cellular ratio of metabolites NAD^+^/NADH activates the deacetylase activity of Sirtuin-1 and destabilizes Foxp3 protein, while acetyl-CoA increases acetylation level of Foxp3 protein and promotes its stabilization. (5) ROS promotes SENP3-driven Bach2 deSUMOylation and nuclear localization, thus stabilizing Foxp3 expression. (6) Short-chain fatty acids stabilize Foxp3 expression, possibly by inhibiting HDAC-mediated suppression of *Foxp3* gene expression and Foxp3 protein deacetylation. (7) Unconstrained activation of mTORC1 and glycolysis inhibit Foxp3 expression and reduce suppressive activity of Treg cells. (8) LKB1 prevents STAT4 activation and binding to CNS2 of *Foxp3* gene, thus preventing the destabilization effect by inflammatory cytokines. LKB1 also regulates Foxp3 expression through activation of mevalonate pathways. RA, all-trans retinoic acid; VDRE, vitamin D response element; CNS2, conserved non-coding sequence 2; NAD, nicotinamide adenine dinucleotide; ROS, reactive oxygen species; HDAC, histone deacetylase.

The molecular mechanisms that prevent the loss of Foxp3 expression have been extensively studied, with the current understanding that Foxp3 expression is maintained through transcriptional, epigenetic and post-translational regulation. First, multiple transcription factors regulate *Foxp3* gene expression by directly binding to *Foxp3* gene promoter, such as STAT5, NFAT, and Foxo1. In addition, the *Foxp3* gene locus contains conserved non-coding sequence (CNS) elements, which recruit transcription factors to regulate gene expression (40–42). For example, CNS1 responds to TGF-β and recruits Smad3 (43); CNS2 recruits STAT5 (35), NFAT (44), RUNX (45), and CREB (46), among others; and the NF-κB signaling component c-Rel binds to CNS3 (47). Second, CNS2 contains a Treg cell-specific demethylated region (TSDR) (48), which is largely demethylated in tTreg cells and partially methylated in iTreg or pTreg cells (41, 42, 49, 50). The demethylated TSDR allows for recruitment of transcription factors, such as Foxp3 itself, CREB, and Ets-1, to stabilize Foxp3 expression (46, 51, 52). Third, acetylation, phosphorylation and ubiquitination have been identified to orchestrate Foxp3 protein stability (42). In particular, recent studies have established a critical role of metabolism in regulating Treg cell stability through interplaying with the established mechanisms of transcriptional, epigenetic, and post-translational control of Foxp3 expression (Figure 1). Below, we summarize the progress in metabolic regulation of Treg cell stability. We first discuss how environmental nutrients and metabolites influence Foxp3 stability. Then, how intrinsic cellular metabolism modulates Treg cell lineage identity is detailed. Finally, the signaling mechanisms for establishing metabolism-dependent control of Foxp3 expression are described.

### Environmental Nutrients and Metabolites

Multiple dietary nutrients, vitamins, and metabolites can directly modulate Foxp3 expression in Treg cells. Among them, the vitamin A metabolite all-*trans* retinoic acid (RA), produced by specific dendritic cell (DC) subsets, directly and indirectly modulates *Foxp3* expression (53–56). RA directly increases the activation of extracellular-related kinase (ERK) signaling to promote *Foxp3* expression (56). RA also increases histone methylation and acetylation of the promoter and CNS at the *Foxp3* gene locus (56). RA indirectly promotes TGF-β-mediated Foxp3^+^ Treg cell conversion by relieving inhibition from CD44^hi^ memory T cells (57). Specifically, CD44^hi^ memory T cells release a series of inflammatory cytokines, such as IL-4, IL-21, and IFNγ, which act synergistically to inhibit TGF-β-induced Foxp3 expression; however, RA suppresses these pro-inflammatory cytokine programs and therefore stabilizes Foxp3 expression (57). RA can also prevent the loss of *FOXP3* expression during human Treg cell expansion and in inflammation, with superior efficacy as compared with rapamycin (an mTORC1 inhibitor) that is known to promote stable Foxp3 expression (55).

In addition to vitamin A, vitamins C and D have been directly linked to the regulation of Foxp3 expression. Recent research has established roles for vitamin C in immune cell function, including its ability to stabilize *Foxp3* expression by demethylation of CNS2 region in iTreg cells (58, 59). Specifically, the CpG motifs of CNS2 in iTreg cells are partially methylated (48, 60), and CNS2 becomes demethylated after treatment with vitamin C, whose effect is mediated by the Tet family demethylase proteins (58, 59). Deletion of Tet2 blocks the demethylation effect of vitamin C (58, 59). Deletion of Tet2/Tet3 in Treg cells indeed leads to unstable Foxp3 expression (61, 62). Vitamin D3 is synthesized in the skin in response to ultraviolet light or acquired from diet, and the vitamin D3 metabolites, 25-dihydroxyvitamin D3 [25(OH)VD_3_] and the active form 1,25(OH)_2_VD_3_, can promote *Foxp3* expression in TCR and IL-2-activated CD4^+^ T cells (63, 64). Subsequent analysis has revealed the presence of vitamin D response element (VDRE) in the intronic conserved CNS region (+1714 to +2554) of the human *FOXP3* gene as a functional enhancer, which underlies how vitamin D3 induces *FOXP3* expression (65). However, the specific transcriptional regulation mechanisms of VDRE on *FOXP3* gene remain to be ascertained.

Other metabolites also regulate Foxp3 expression in Treg cells, such as those from tryptophan and purine metabolism. DCs that express the enzyme indoleamine 2,3-dioxygenase (IDO) can catabolize tryptophan. The IDO-dependent catabolic program subsequently induces Foxp3^+^ Treg cell generation through inhibition of IL-6 production by DCs (66, 67). Mice treated with the tryptophan metabolite 3-hydroxyanthranilic acid (3-HAA) increase expression of TGF-β in DCs, display increased percentage of Treg cells, reduced frequencies of Th1 and Th17 cells, and ameliorated development of experimental autoimmune encephalomyelitis (EAE) (68). Tryptophan also serves as the precursor for *de novo* nicotinamide adenine dinucleotide (NAD^+^) synthesis (69), which is also regenerated from NADH by reduction of pyruvate to lactate during activation of glycolysis. The NAD^+^/NADH ratio directly regulates the activity of deacetylase Sirtuin-1 (70). Since acetylation improves Foxp3 protein stability (71), Sirtuin-1 post-translationally impairs the acetylation and stability of Foxp3 (72). Foxp3 expression is also regulated by metabolites from extracellular purine metabolism, which orchestrates the balance of proinflammatory ATP and anti-inflammatory adenosine. Extracellular ATP and NAD^+^ that are released by cell lysis or non-lytic mechanisms during cell damage and inflammation can activate the P2X7 receptor and induce T cell death. Treg cells highly express P2X7 receptor, which upon activation can limit Foxp3 stability and enhance Treg cell conversion into Th17 cells (73). Intriguingly, Treg cells express high levels of the ectonucleotidases CD39 and CD73 on the surface, which convert excess extracellular ATP into immunosuppressive adenosine to relieve the harmful effect of extracellular ATP and increase their suppressive function (74). The results suggest that purine metabolism could be important for Treg cells to maintain stability. Overall, we still know little about how nutrients and metabolites regulate Treg cell stability, especially *in vivo*, which may be uncovered by using metabolomics techniques.

### Cellular Metabolism

Cellular metabolism is also closely related to Treg cell stability. Compared to Th1, Th2, and Th17 cells, Treg cells are less reliant on glycolysis and use mitochondrial metabolism and oxidative phosphorylation (OXPHOS) for energy production (16). *In vitro* studies reveal that expression of Foxp3 reprograms T cell metabolism by suppressing glycolysis and enhancing OXPHOS (17, 18). The effector molecules CTLA4 and PD-1 on Treg cells also suppress glycolysis in T cells (75, 76). Several studies have indicated that elevated glycolysis may be detrimental to Treg cell induction, as inhibition of glycolysis promotes the induction of Foxp3 expression in response to TGF-β and IL-2 stimulation (77, 78). In addition, deletion of HIF-1α, a transcription factor that can promote glycolysis, also leads to increased Foxp3 induction (78). *In vivo*, transgenic mice expressing Glut1 (Glut1-tg) have a greater proportion of CD25^lo^Foxp3^+^ cells than those from wild-type mice (18). Further analysis has demonstrated that Glut1-tg expression reduces Foxp3 expression in iTreg cells and *in vivo* during intestinal inflammation. Analysis of key metabolism-related proteins has also illustrated that excessive glycolysis can lead to reduced Treg cell stability. For instance, c-Myc promotes glycolysis in T cells (79), whose activity was recently shown to be inhibited by autophagy to maintain Treg cell stability (25). Furthermore, specific deletion of phosphatase and tensin homolog (PTEN) in Treg cells also leads to PI3K/Akt-mediated hyperactivation of glycolysis, a greater proportion of CD25^lo^Foxp3^+^ cells similar as Glut1-tg mice, and increased methylation of the TSDR region of *Foxp3* gene (26, 27). These studies together demonstrate that unrestrained glycolysis results in reduced Treg cell stability. Of note, a recent study in human Treg cells has demonstrated that the glycolytic enzyme Enolase-1 binds to the *FOXP3* promoter and its CNS2 region, and represses the transcription of a splice isoform containing Exon-2 (FOXP3-E2), which is important for Treg cell suppressive activity (80). Glycolysis drives Enolase-1 to translocate out of nucleus and relieves the repression of transcription of FOXP3-E2 (80). Moreover, recent studies have established that glycolysis also increases Treg cell migration (81). It is reasonable to speculate that Treg cells precisely calculate and balance cellular glucose consumption, where heightened glycolysis increases Treg cell proliferation or migration to fill the niche and expand the pool *in vivo*, but this activity is balanced by other metabolic programs (for example, OXPHOS) to maintain lineage stability and suppressive activity. Intriguingly, TLR signals in Treg cells have been shown to promote PI3K/Akt signaling, and increase glycolysis and proliferation, while reducing suppressive function (18). Thus, we propose that Treg cell stability and function are under precise control of glycolysis. However, the detailed mechanisms whereby glycolysis interplays with Foxp3 expression remain to be ascertained.

As noted above, several recent studies have demonstrated crucial roles for mitochondrial metabolism and OXPHOS for Treg cell suppressive activity both at steady state and in the tumor microenvironment (82–85). Treg cells display greater mitochondrial mass and higher levels of reactive oxygen species (ROS) compared with conventional T cells (82), likely produced from OXPHOS. ROS is reported to increase the SUMO-specific protease 3 (SENP3) stabilization, and trigger Bach2 deSUMOylation (86). DeSUMOylation of BACH2 prevents its nuclear export, thus maintaining Foxp3 expression and Treg cell stability (86). Moreover, Treg cell-specific deletion of mitochondrial transcription factor A (Tfam) (promotes synthesis of mitochondrial DNA-derived proteins), can destabilize Foxp3 expression, which is associated with enhanced methylation in the TSDR of *Foxp3* locus in Treg cells under inflammatory contexts (87), whereas Tfam is dispensable for Foxp3 stability in the absence of inflammation (85). These findings are in agreement with a recent study that showed specific deletion of mitochondrial complex III impairs suppressive function without altering Foxp3 expression in Treg cells (84). Thus, OXPHOS in Treg cells seems to enforce Treg cell function independently of regulating their stability.

A growing area of interest is the regulation of OXPHOS by extracellular nutrients in Treg cells. As upregulation of glycolysis can be detrimental to Treg cell function, several studies have instead focused on the roles of fatty acids and FAO for the regulation of Treg cell function and stability. FAO involves the degradation of fatty acids by the sequential removal of two-carbon units from the acyl chain to produce acetyl-CoA, which enters the mitochondrial tricarboxylic acid (TCA) cycle to regulate mitochondrial OXPHOS and other functions. Short-chain fatty acids can indeed stabilize Foxp3 expression, possibly by inhibiting the expression of histone deacetylases (HDACs), such as HDAC6 and HDAC9 that can destabilize Foxp3 protein stability (88). However, the roles of HDACs is likely complex, as they may also orchestrate acetylation status of other transcription factors, like STAT5 that induces and sustains *Foxp3* gene expression (89). Other studies have also revealed that acetyl-CoA levels contribute to Foxp3 expression through the post-translational control of protein acetylation and protein stability in Treg cells (90). However, FAO may serve only context-dependent roles in Treg cells. Carnitine palmitoyl-transferase 1A (Cpt1a) is a protein found in the outer mitochondrial membrane that catalyzes the esterification of long-chain acyls with carnitine to form acyl-carnitine and is considered to be the rate-controlling for long-chain FAO. The frequency and total number of Treg cells in mice lacking *Cpt1a* in CD4^+^ cells or in Foxp3^**+**^ Treg cells are comparable in different tissues (91). *Ex vivo*-isolated Treg cells lacking *Cpt1a* display normal Foxp3 expression levels and similar mitochondrial oxidative capacity relative to their wild-type counterparts. As the study is unable to rule out the possible role of medium-chain and short-chain fatty acids, the effects of fatty acid metabolism on Foxp3 expression and stability require further investigation.

### Metabolic Signaling

Several signaling pathways that impact T cell metabolism have been identified. Among them, PI3K/Akt and LKB1/AMPK (AMP-activated protein kinase) signaling pathways play central roles. PI3K catalyzes the conversion of PtdIns-4,5-P_2_ (PIP2) toward PtdIns-3,4,5-P_3_ (PIP3) and activates kinases with Pleckstrin homology (PH) domains, most notably Akt (92). PI3K/Akt signaling is activated by upstream TCR and IL-2 signaling in Treg cells (93). Akt also directly phosphorylates the Foxo (Foxo1 or Foxo3a) transcription factors and blocks their nuclear translocation (94). Stable tTreg cells display hypoactivation of Akt, resulting in enhanced nuclear Foxo abundance on the promoter regions of *Foxp3*, leading to stable Foxp3 expression (95–97). These observations are consistent with those showing that Foxo activity limits glycolysis in T cells via impairing c-Myc function (98). Ligation of neuropilin-1 by Sema4a promotes Treg cell stability through PTEN-dependent inhibition of Akt activity (99), which facilitates Foxo nuclear localization and thereby increases Foxp3 expression (99). These results indicate an indispensable role of the Akt/Foxo pathway in orchestrating Treg cell stability.

Akt can also indirectly affect the activation of the mTOR complexes, mTORC1 and mTORC2, which integrate upstream metabolic signals for metabolic programming (100). Akt phosphorylates TSC2 to relieve TSC complex inhibition on mTORC1, which is a critical driver of glycolysis through augmenting the expression of glucose transporters, such as Glut1, or transcription factors, including c-Myc (100, 101). mTOR inhibition by rapamycin drastically enhances TGF-β-induced Foxp3 expression *in vitro* (102), indicating that Treg cells require low mTORC1 activity for induction of Foxp3 expression. However, deletion of mTORC1 in Foxp3^+^ Treg cells does not affect Foxp3 expression on a per cell basis *in vivo* (22), whereas conditional deletion of TSC1 in Treg cells leads to impaired Foxp3 expression and heightened IL-17 production under inflammatory conditions (103). Thus, unconstrained mTOR activation can have deleterious impacts on Treg cell stability. Several studies have also investigated the role of mTORC2 in Treg cell stability and function. Rictor (the obligate protein for mTORC2) deletion in Treg cells results in no obvious abnormalities (22). Rictor-deficient T cells also retain their capacity to become Foxp3^+^ iTreg cells (104, 105). Thus, mTORC2 has a less dominant function *in vivo* and *in vitro* than that of mTORC1 at steady state. However, upon Foxp3 deficiency, mTORC2 is responsible for augmented aerobic glycolysis and OXPHOS in Treg cells, and deletion of Rictor could restore the suppressive activity of Foxp3-deficient Treg cells (106). TCR and IL-2 are two of the most important upstream drivers for mTORC1 activation in Treg cells. Co-stimulation with TCR and IL-2 *in vitro* can relieve the suppression of PTEN on Akt-mTOR activation in Treg cells, reverse the anergic state of freshly-isolated Treg cells and promote their proliferation (107). How Treg cells maintain mTOR activation-mediated expansion and prevent unconstrained mTOR activation-induced instability *in vivo* warrants further investigation. However, it should be noted that hormones, such as leptin, may allow for temporal tuning of mTOR activation to promote appropriate expansion of Treg cells without affecting Foxp3 stability (108). Thus, a key future direction will be to dissect the upstream metabolic inputs that promote mTOR activity in Treg cells, toward understanding the effect of environmental cues and the mechanisms by which they are transmitted to impact Treg cell function.

The AMPK pathway, which is activated in response to cellular stress (e.g., AMP/ATP ratio), suppresses mTOR signaling and promotes mitochondrial OXPHOS rather than glycolysis (109–111). The AMPK agonist AICAR (5-aminoimidazole-4-carboxamide ribonucleotide) strongly enhances Treg cell expansion (112). Activation of AMPK signaling by metformin-induced FAO also promotes Treg cell generation *in vivo* (16). iTreg cells have high levels of activated AMPK and FAO (16), likely due to the ability of AMPK to modulate Cpt1a activity and increase fatty acid import into mitochondria for β-oxidation (110). The serine-threonine kinase LKB1 is activated in response to TCR signals and can directly phosphorylate and activate AMPK (111, 113). LKB1 promotes mitochondrial fitness and FAO in Treg cells (113); however, these events appear to be AMPK-independent (113–116), suggesting other downstream LKB1 targets as important regulators of metabolic programming in Treg cells. In addition, recent analysis of LKB1-deficient Treg cells demonstrates that LKB1 enhances Foxp3 expression by preventing CNS2 methylation (114), and by activation of the mevalonate pathway that generates many metabolites, including cholesterol and the isoprenoid geranylgeranylpyrophosphate (GGPP) (115). Specifically, LKB1 prevents STAT4 activation and binding to CNS2, thus maintaining Foxp3 stability in response to STAT4-inducing inflammatory cytokines (114). Moreover, mevalonate or GGPP treatment restores the function and stability of LKB1-deficient Treg cells (115). The precise mechanisms that control AMPK and LKB1 activation in Treg cells require further study.

## Shaping Treg Cell Plasticity by Metabolism

Unlike loss of stability, “plastic” Treg cells tend to retain Foxp3 expression, but acquire the expression of transcription factors associated with effector T cell programs (called Th-like Treg cells). Plasticity is essential for Treg cells to exert specific suppressive activity toward selective types of inflammation and various environmental conditions. Specifically, Treg cells express the transcription factor T-bet to suppress type-1 inflammation, and acquire IRF4 and STAT3 to inhibit type-2 and type-17 inflammation, respectively (117–120). Gata3 expression by Treg cells is also important for suppression of type-2 inflammation in barrier sites like the skin and intestine (121, 122). In other studies, loss of Gata3 in Treg cells is associated with reduced Foxp3 expression and increased expression of RORγt during inflammation (123, 124), while co-deletion of T-bet and Gata3 directly leads to severe autoimmune-like disease and impaired Treg cell function (125). Moreover, Bcl6 is induced to generate T-follicular regulatory (Tfr) cells, which control germinal center responses (126–128). It is important to note that subsequent studies have revealed that these Th-like Treg cells may display impaired suppressive function in certain contexts (129–132). Th-like Treg cells normally display demethylated state on Foxp3 TSDR region (129). However, in certain inflammatory contexts, the epigenetic status could be altered by the presence of environmental cytokines, which may allow these cells to express proinflammatory cytokines (132). Thus, it remains unclear why Th-like Treg cells are suppressive in some contexts but pro-inflammatory in others. Notably, there is considerable controversy about whether instability is a separate cellular state from plasticity (33, 34). Both instability and plasticity have been observed in various autoimmune diseases in mice and humans, but the mechanistic connection between instability and plasticity in disease settings remains incomplete. Whether Th-like Treg cells represent a transient stage toward becoming Foxp3^−^ Treg cells requires further investigation.

The best characterized Th-like Treg cells are Th1-like Treg cells, which express T-bet and CXCR3 (117). Th1-like Treg cells have been observed in autoimmune (Sjögren syndrome) (133) and tumor models. In fact, increased expression of IFN-γ by Treg cells can markedly improve checkpoint blockade therapy (134, 135). The dominant effect of IFN-γ^+^ Treg cells in these tumor models indicates that these cells not merely lose suppressive function but gain anti-tumor effector activity. In humans, IFN-γ^+^ Treg cells are found in patients with relapsing/remitting multiple sclerosis (RRMS), type 1 diabetes and autoimmune hepatitis (129, 132, 136). *In vitro*, IL-12 stimulation induces T-bet, CXCR3, CCR5, and IFN-γ expression in Treg cells, which maintain a demethylated TSDR and *FOXP3* expression (129, 131). These results underscore a critical role of IL-12 signaling in promoting Th1-like Treg cell generation. The observations are consistent with those that T-bet expression is further induced in type-1 inflammation, but studies using mouse models have shown that T-bet and IFN-γ are not co-expressed in Th1-like Treg cells as Th1 cells, possibly due to delayed induction of IL-12 receptor component IL-12Rβ2 and IL-12 signaling (117). Thus, how IL-12 can differentially regulate Th1-like programming in Treg cells as compared with Th1 cells remains an interesting question to address.

**Figure 2:**
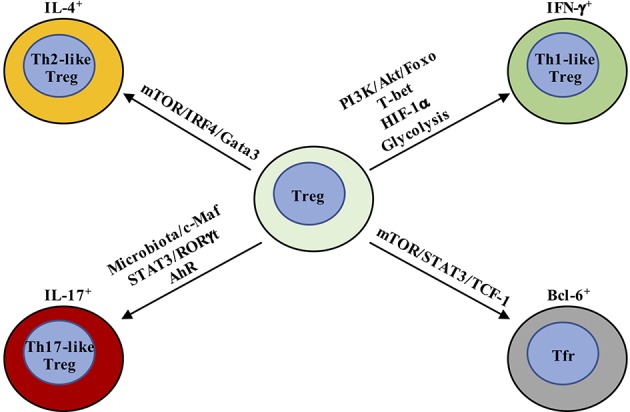
Metabolic and transcriptional regulation orchestrates Treg cell plasticity. Th1-like Treg cells are T-bet^+^ and express IFN-γ, whose differentiation is driven by IL-12-mediated PI3K/Akt/Foxo activation. In addition, HIF-1α increases IFN-γ expression by enhancing glycolysis and directly binding *Ifng* gene to promote Th1-like Treg cell differentiation. Th2-like Treg cells are characterized by the expression of Gata3 and IRF4 and production of IL-4 and IL-13. TCR and downstream mTOR activation promote IRF4 expression to facilitate Th2-reprogramming in Treg cells. Th17-like Treg cells express RORγt in addition to Foxp3. IL-6 and downstream STAT3 activation are critical for the generation of IL-17-producing Treg cells. The tissue in which Th17-like Treg cells have been best characterized is the intestine, where Treg cells adapt to the local environmental cues, like microbiota and AhR ligands. T-follicular regulatory (Tfr) cells co-express Bcl-6 and Foxp3. mTORC1 promotes the phosphorylation of STAT3 and induces the expression of TCF-1 to drive Bcl-6 expression.

Metabolic pathways are emerging regulators of Treg cell plasticity (Figure 2) (95, 113, 131, 137). For instance, PI3K/Akt/Foxo signaling plays an important role in regulating IL-12-induced IFN-γ production in Treg cells (131), but also promotes metabolic programming as discussed below. Freshly-isolated IFN-γ^+^ Treg cells display increased expression of Akt1 and decreased expression of Akt2 and PTEN (131). *In vitro*, IL-12 directly activates PI3K/Akt/Foxo pathway to drive Th1 polarization in Treg cells (131). Moreover, Foxo1 can be recruited to a regulatory element upstream of the transcriptional start site of *Ifng* gene. Treg cell-specific deletion of Foxo1 leads to upregulation of *Ifng* gene expression and increased IFNγ^+^ Treg cells (95, 96). Notably, the suppressive activity of Foxo1-deficient Treg cells is mostly corrected by blockade of PI3K/Akt pathways (95). As described above, decreased Foxo activity is correlated with increased glycolysis in T cells (98), and increased glycolysis is recently reported to promote Th1 cell differentiation by epigenetic regulation of *Ifng* gene locus (138), suggesting that Treg cells may also adopt similar interplay between metabolic and epigenetic regulation to polarize Th1-like differentiation. In further support of this notion, a recent study has reported that deficiency of the E3 ubiquitin ligase VHL causes Treg cells to adopt an IFN-γ^+^ Th1-like Treg cell program (139). This event is mediated through both a shift in glycolytic programming driven by HIF-1α, and increased binding of HIF-1α to the *Ifng* gene, further strengthening the link between glycolysis and IFN-γ^+^ Treg cell generation. Whether metabolites or key glycolytic enzymes promote the generation of Th1-like Treg cells needs further investigation.

For Th2-like Treg cells, both aberrant and beneficial phenotypes have been observed. Toward the former, programming of Th2-like Treg cells is associated with compromised suppressive function in respiratory syncytial virus (RSV) infection and food allergy mouse models (140, 141). Treg cell-specific deletion of E3 ubiquitin ligase Itch also leads to generation of Th2-like Treg cells that can drive Th2-related pathologies (142). As noted above, Th2-like Treg cells are characterized by expression of Gata3 and IRF4, as well as production of IL-4 and IL-13 (140–142). Th2-like programming can be induced by IL-4R signals that promote Gata3 expression (140, 141). Additionally, TCR signals induce IRF4 expression in Treg cells, and mice with Treg cell-specific deletion of IRF4 develop type-2 inflammation, as IRF4 expression suppresses expression of pro-inflammatory Th2-specific genes in Treg cells (119). IRF4 can also form complex with Foxp3 to regulate the transcriptional program and generation of effector Treg (eTreg, CD44^+^CD62L^−^) cells (119, 143). mTOR functions downstream of TCR to drive IRF4 expression to control Th2 inflammation (85). Moreover, defects in metabolic reprogramming caused by LKB1 deletion in Treg cells are associated with impaired suppression of Th2 immunity (113). These results together suggest that metabolic pathways may have a great impact upon Th2-like Treg cells, although the precise mechanisms have not been defined.

Th17-like Treg cells co-express RORγt with Foxp3 (144, 145) and can be generated in the periphery (145). Also, under inflammatory conditions, RORγt^+^ Treg cells can be generated from tTreg cells (146). These IL-17-producing Treg cells retain suppressive function (144, 145, 147). IL-6 and downstream STAT3 activation, as well as IL-23, IL-1β, and IL-21, are critical for the generation of IL-17-producing Treg cells (148–150). The tissue in which Th17-like Treg cells have been best characterized is the intestine, where Treg cells adapt to the local environmental changes through functional and phenotypic reprogramming as discussed more below. In addition, as a central coordinator of metabolism in T cells, mTOR also controls Treg cell polarization into other functionally plastic subpopulations, such as Tfr cells in the germinal center (137). Mechanistically, mTORC1 promotes the phosphorylation of the transcription factor STAT3 and induces the expression of the transcription factor TCF-1 to drive Bcl-6 expression. These results together indicate that metabolism plays a critical role in modulating Treg cell plasticity. However, the limitations of low cell numbers and inability to isolate Th-like Treg cells based on surface markers impede analysis of how metabolism controls their homeostasis and function. The development of single cell metabolomics (151, 152) and new fate-mapping genetic tools (118, 125) could help understand the metabolic regulation of Th-like Treg cells in the future.

## Metabolic Adaption in Tissue Treg Cells

**Figure 3:**
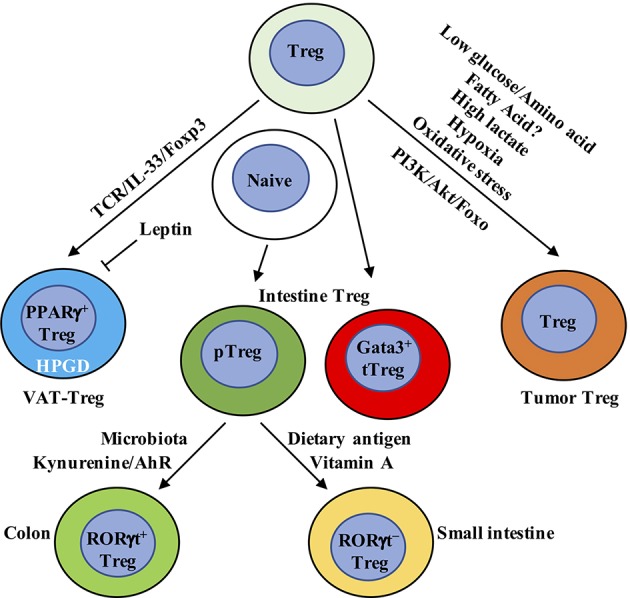
Metabolic and transcriptional adaption in tissue Treg cells. VAT-Treg cell accumulation is dependent upon TCR, Foxp3, and IL-33 signaling. VAT macrophages and DCs are the major antigen-presenting cells that express MHC-II to activate and increase VAT-Treg cells. In VAT, white adipose tissue produces IL-33 to increase VAT-Treg cell generation. Leptin produced by adipose cells inhibits VAT-Treg cell proliferation and induces anergy. Leptin-neutralizing antibody reverses anergy and increases mTOR activation and Treg cell proliferation. VAT-Treg cells have high expression of enzyme hydroxyprostaglandin dehydrogenase (HPGD), which is dependent on PPARγ. HPGD produces 15-keto PGE_2_ to suppress conventional T cell activation and proliferation. Intestinal Treg cells mainly contain Gata3^+^ tTreg, RORγt^+^, and RORγt^−^ pTreg cells. RORγt^+^ pTreg cells are the dominant population in the colon, while RORγt^−^ pTreg cells mainly localize in small intestine. Their difference is mainly derived from the dominant effect of microbiota and dietary metabolites, respectively, in the colon and small intestine. In the colon, metabolism of tryptophan produces AhR ligands, such as kynurenine, to selectively enhance the generation of Foxp3^+^ Treg cells. In the small intestine, dietary vitamin A is present at high concentrations. RA, a metabolite from vitamin A, induces pTreg cells from naïve T cells in the small intestine in combination with TGF-β. RA also induces the expression of CCR9 on the surface of Treg cells to facilitate their migration to the small intestine. In tumor tissue, Treg cells not only display eTreg cell phenotypes similar to non-lymphoid organs but also express some tumor-specific gene signatures. Compared to conventional T cells, Treg cells have a metabolic advantage in tumor microenvironment, which has low glucose/amino acid, high lactate/hypoxia, and elevated oxidative stress. Genetic or pharmacological perturbation of metabolic pathway PI3K/Akt/Foxo signaling directly impacts tumor-resident Treg cell homeostasis, function and anti-tumor immunotherapy. VAT, visceral adipose tissue; AhR, aryl hydrocarbon receptor.

An emerging concept is that Treg cells found in non-lymphoid organs, including adipose tissue, intestine, and tumors, display marked tissue-specific heterogeneity. Tissue-specific transcriptional programs, possibly orchestrated by unique transcription factors, such as PPARγ, RORγt, and Gata3, facilitate the ability of tissue-resident Treg cells to maintain tissue homeostasis. Non-lymphoid and tumor tissues have dramatic differences in their metabolic environments than secondary lymphoid organs, thus suggesting differences in their nutrient and intracellular metabolic requirements. Notably, Treg cells in non-lymphoid organs and tumor tissues are largely eTreg cells. mTOR has been identified as a critical driver for the generation and function of eTreg cells in both lymphoid and non-lymphoid tissues (85, 153), which acts downstream of TCR signals to drive IRF4 expression and mitochondrial metabolism, facilitating eTreg cell generation. The result further underscores a central role of metabolism in modulating tissue Treg cell heterogeneity and homeostasis (Figure 3).

### Adipose Tissue

Among different types of adipose tissues, visceral adipose tissue (VAT) is enriched for Treg cells (hereafter called VAT-Treg cells) whose major nutrient source seems to be fatty acids derived from the environment. VAT-Treg cells display a distinct gene expression signature compared to Treg cells from secondary lymphoid organs (154). Peroxisome proliferator-activated receptor gamma (PPARγ) expression is critical for establishing the unique VAT-Treg cell transcriptional program and homeostasis (155). Indeed, mice that specifically lack PPARγ in Treg cells have a significantly reduced population of VAT-Treg cells, but not Treg cells in other organs. The increased expression of PPARγ promotes fatty acid metabolism and stimulates the accumulation and suppressive activities of the VAT-Treg cells (155). Moreover, VAT-Treg cells express CD36, a receptor that facilitates uptake of long-chain fatty acids and contributes to lipid accumulation in contexts of high-fat diets and obesity (155). Obese mice display significantly decreased VAT-Treg cells, which may be partly explained by the heightened leptin signaling (108, 156). Leptin may be produced by adipose cells (157). VAT-Treg cells express leptin receptor, receive increased leptin secretion from VAT of obese mice and decrease their proliferation and number, suggesting that leptin may be an important environmental cue in the adipose tissue for modulating Treg cells (21). This observation is consistent with other studies that identified a central role of leptin in inhibiting Treg cell proliferation and resulting in Treg cell anergy *in vitro* (108, 156). In addition, a recent report has found that the enzyme hydroxyprostaglandin dehydrogenase (HPGD) is highly expressed in VAT-Treg cells, which is dependent on PPARγ. HPGD catabolizes prostaglandin E_2_ (PGE_2_) into the metabolite 15-keto PGE_2_ to suppress conventional T cell activation and proliferation (158). These results together demonstrate that Treg cells adapt to unique VAT environments for their homeostasis and function.

Recently, using a T cell receptor transgenic mouse line in which VAT-Treg cells are enriched, the Mathis group identified a two-step, two-site developmental axis for VAT Treg cells (159). Single cell RNA-sequencing (scRNA-seq) and assay for transposase-accessible chromatin sequencing (ATAC-seq) analyses reveal that splenic Treg cells that are destined to become VAT-Treg are primed within the secondary lymphoid organs, where they are defined by low expression of PPARγ that allows them to upregulate selective VAT-Treg-associated transcriptional programs. These PPARγ^lo^ Treg cells then migrate to the VAT, where they are educated and exposed to local microenvironmental cues to differentiate into VAT-Treg cells. The accumulation of VAT-Treg cells is dependent upon TCR, Foxp3, and IL-33 signaling, and is associated with substantial epigenetic remodeling. Specifically, the critical role of IL-33 in the development and maintenance of VAT-Treg cells is dependent upon the high expression of IL-33 receptor ST2 (160). IL-33 treatment leads to reduced obesity, improved glucose tolerance, and increased proportion of ST2^+^ Treg cells in the VAT of genetically obese diabetic mice (161). White adipose tissue is the main source of IL-33 in VAT (162), and γδ T cells may also promote IL-33 production by VAT, as mice lacking γδ T cells exhibit decreases of ST2^+^ Treg cells in VAT (163). Subsequent studies also demonstrate that IL-33 is a critical driver for the differentiation and function of other tissue-resident Treg cells, including in the brain (164) and intestine (165). Moreover, TCR: MHC-II interactions are also required for the development and maintenance of VAT-Treg cells (166). In MHC-II-deficient mice, VAT-Treg cell number is greatly reduced, associated with less expression of Gata3 (160). Among the antigen-presenting cells that express MHC-II, VAT macrophages (167) and DCs (160) have been reported to activate and increase VAT-Treg cells. These results together demonstrate that the ligand-receptor interactions between cells in the adipose tissues instruct plasticity in the VAT and generate unique VAT-Treg cells. However, questions regarding the phenotype and function of VAT-Treg cells in humans and how to manipulate VAT-Treg cells to prevent obesity and related metabolic diseases still remain to be addressed.

### Intestine

The intestine is constituted by small intestine and large intestine, with the latter containing cecum and colon. Due to the direct exposure to exogenous dietary antigens, the intestine must both protect the host from harmful pathogens and maintain tolerance to intestinal microbiota and beneficial metabolites. This sophisticated task requires a close collaboration within the intestinal network to recognize and distinguish the harmful and beneficial material from the diet. The intestinal wall contains four major layers: mucosa, submucosa, muscularis, and serosa (168). The epithelial cells of the mucosa form the first physical barrier and are supported by an underlying network of immune cells in the lamina propria, local lymphoid structure like Peyer's patches (PPs) and the draining lymph nodes. Treg cells play a critical role in suppressing local inflammatory responses and maintaining intestinal homeostasis (169). To adapt to the highly specific environment, Treg cells develop tissue-specific heterogeneity. Indeed, a substantial proportion of Helios^−^neuropilin-1^−^ pTreg cells are found in the small intestine and colon, as well as Helios^+^neuropilin-1^+^ tTreg cells that express Gata3 (170). Thus, the intestine represents a unique mucosal site that requires both tTreg and pTreg cells for homeostasis.

The cues that allow for pTreg cell induction in the intestine have been extensively studied. Due to the similar differentiation requirements and expression of RORγt, RORγt^+^Foxp3^+^ Treg cells were once thought to be precursors for unstable Treg cells that would differentiate into Th17 cells (171, 172). However, a large population of RORγt^+^ pTreg cells are present in the colon under steady state (170, 173, 174), while the small intestine mainly contains RORγt^−^ pTreg cells (175). Transcriptomic analysis has revealed that the signature of RORγt^+^Foxp3^+^ Treg cells is similar to both Treg and Th17 cells, but has a higher overlap with Treg cells (174). Further, RORγt^+^Foxp3^+^ Treg cells display significantly demethylation at Treg cell-specific signature genes like *Foxp3, Ctla4*, and *Tnfrsf18*, indicating that they are a lineage-stable population with potent suppressive activity (174). TCR repertoire analysis further reveals that the RORγt^+^Foxp3^+^ population is distinct from other colonic T cell subsets (176). The development of RORγt^+^Foxp3^+^ cells in the mucosa can be derived from naïve CD4^+^ T cells in the periphery and pass through a RORγt^−^ Treg intermediate before co-expressing RORγt (176). CX3CR1^+^ antigen-presenting cells may provide specific signals for the development of RORγ^+^ Treg cells (176). Moreover, loss of RORγt^+^Foxp3^+^ Treg cells exaggerates several models of mucosal autoimmunity, indicating that this subpopulation is functionally important (170, 173, 174). Thus, co-expression of RORγt and Foxp3 is also essential for establishing Treg cell function in the intestine.

The intestinal microenvironment differs in the colon and small intestine. The colon harbors multiple species of microbiota to facilitate the development of RORγt^+^ Treg cells (170, 173). Germ-free mice treated with antibiotics greatly diminish the abundance of colonic pTreg cells, leaving a predominant Helios^+^ tTreg cell population (173, 177, 178). The abundance of RORγt^+^ Treg cells is linked to changes in the microbiota, whereas RORγt^−^ Treg cells are relatively insensitive (170, 173, 175, 179). Many microbial species such as *Clostridium ramosum, Staphylococcus saprophyticus, Bacteroides thetaiotaomicron*, and *Clostridium histolyticum*, have the potential to induce pTreg cell generation (170, 173, 180–183). In addition, *Helicobacter hepaticus*-reactive naïve CD4^+^ T cells differentiate into RORγt^+^ Treg cells upon *H. hepaticus* colonization, which is dependent upon the transcription factor c-Maf (183). The RORγt^+^ Treg cell differentiation is also dependent upon constant antigen exposure, as mice deficient in MHC-II have greatly fewer RORγt^+^ Treg cells (170). The aryl hydrocarbon receptor (AhR) is a sensor that detects environmental pollutants and physiological compounds from diet, host cells, and microbiota (184). Microbiota, such as *Lactobacillus* species, metabolize tryptophan to generate AhR ligands (185). Metabolism of tryptophan produces a series of AhR ligands, such as kynurenine, to selectively enhance the generation of Foxp3^+^ Treg cells *in vitro* (186). Intriguingly, the microbiota-induced RORγt^+^ Treg cells express the highest amount of AhR compared to various organs (179). Treg cell-specific deletion of AhR impairs pTreg, but not tTreg, cell homeostasis in the colon (179). Moreover, AhR-expressing Treg cells have enhanced *in vivo* suppressive activity compared with Treg cells lacking AhR expression in a T cell transfer model of colitis, highlighting a central role of AhR in microbiota-induced pTreg cell generation and function (179). Microbiota also increase the expression of GPR15, an orphan G-protein coupled receptor, to promote the specific homing of Treg cells to the large intestine (187). Therefore, microbiota promote the generation, function, and influx of Treg cells in the large intestine.

Studies comparing changes in dietary composition and germ-free mice indicate that the induction of Treg cells in the small intestine is mainly dependent upon dietary antigens rather than microbiota, and diet-generated Treg cells are mostly pTreg cells (175, 182, 188). For example, all-*trans* RA acts in concert with TGF-β for pTreg cell generation in the small intestine (53, 189, 190), where dietary vitamin A is abundant (191) and is converted to RA with the help of local epithelial cells and DCs (192). Moreover, RA induces the expression of CCR9 on Treg cells, which is required for their migration to small intestine (53, 189). RA-induced pTreg cell generation underlies oral tolerance, as mice treated with a vitamin A-deficient diet display defective in the induction of oral tolerance (193). Recent studies have also indicated a critical role for isoleucine in regulating Treg cell homeostasis in the small intestine (194). Other dietary mechanisms controlling the homeostasis of small intestinal Treg cells *in vivo* require further investigation.

### Tumor Tissue

Tumor tissues are complex environments that contains tumor cells, stromal cells, and infiltrating immune cells (195–197). Due to the malignant cell proliferation, tumor cells require sufficient energy supply and exert metabolic effects on local tumor microenvironment (198). A large population of Treg cells exist in the tumor microenvironment and present a major obstacle for effective anti-tumor therapy (195, 196). Treg cells isolated from tumors are often in an activated state with a metabolic signature that is distinct from lymphoid tissue Treg cells. Their transcriptome shares high similarities with “tissue Treg cells” residing in non-lymphoid tissues (199). Recent studies have identified an important role of Foxo1 in regulating eTreg cells in tumor (200). Hyperactivation of Foxo1 preferentially depletes eTreg cells, which enhances CD8^+^ T cell function and anti-tumor immunity (200), suggesting that targeting PI3K/Akt/Foxo pathway in Treg cells could possibly break the Treg cell barrier in anti-tumor therapy. Inactivation of PI3K p110δ in Treg cells also unleashes cytotoxic CD8^+^ T cells and induces tumor regression and better survival (201–203). These data reveal that subtle perturbations in metabolic signaling could impact tumor-resident Treg cell homeostasis and function. Despite the similarities with Treg cells in non-lymphoid organs, tumor-resident Treg cells also highly express unique signatures, such as *Ccr8, Tnfrsf8, Cxcr3*, and *Samsn1* (199, 204), which might serve as valuable targets for tumor immunotherapy.

Tumor cells modulate several environmental cues to affect tumor-resident Treg cell generation and function, in which the competition of extracellular nutrients stands out. Cancer cells undergo a transition of OXPHOS to aerobic glycolysis, known as the Warburg effect (198). This metabolic shift in cancer cells leads to the consumption of environmental glucose and glutamine, thus leading local T cells to adopt unresponsive or functionally exhausted states (205, 206). In contrast, glucose deprivation can drive Foxp3 expression, shifting T cell differentiation from conventional T cells toward iTreg cells (16, 78, 207). Likewise, tumor cells also compete with T cells for extracellular amino acids for metabolism. Amino acids, especially glutamine and leucine, are required to fuel mTORC1 activation in conventional T cells, promoting T cell differentiation toward Th1, Th2, and Th17 cells, but have a less effect on iTreg cells (208). Deletion of amino acid transporters ASCT2 and SLC7a5 (LAT1) leads to normal Treg cell differentiation (209, 210). Moreover, a decrease of intracellular αKG, caused by the limited availability of extracellular glutamine, also promotes the generation of iTreg cells rather than Th1 cells (211). Interestingly, Treg cells could further deplete environmental amino acid levels by inducing amino acid-consuming enzymes in DCs, such as arginase 1, histidine decarboxylase, or threonine dehydrogenase, through TGF-β and IL-10 secretion. Enzymatic consumption of amino acids by DCs could further inhibit mTORC1 activation, thus synergizing with TGF-β for Treg cell induction (212). These results indicate that nutrient availability in the tumor microenvironment can suppress T cell responses, in part, through induction of Treg cells.

Apart from glucose and amino acids, tumor cells also perturb environmental fatty acid concentrations. In some cancer cells, the extracellular liberation of free fatty acids from more complex lipid species is increased (198). However, the exact regulation of fatty acid availability and diversity in the tumor microenvironment remains elusive and debatable. Mouse iTreg cells are reported to preferentially use FAO (16), and short chain fatty acids can promote iTreg cell differentiation (88, 213). In brain tumors, higher percentages of Treg cells that express CD36 and SLC27A1 (fatty acid transporters) are found (83). Inhibition of lipid uptake with sulfo-N-succinimidyl oleate (SSO) or FAO with etomoxir (Eto) prevents Treg cell immunosuppressive capabilities in this environment (83). Therefore, future studies could analyze if Cpt1a deficiency affects specific tumor Treg cell responses (91). Under steady state, Treg cells rely on mTORC1-mediated lipogenesis for their expansion and function (22), and proliferating Treg cells in the MC38 tumor microenvironment also rely on *de novo* fatty acid synthesis to accumulate intracellular lipids and complement glycolysis for Treg cell expansion (214). Therefore, whether nutrient competitiveness influences and whether fatty acids are preferentially catabolized or synthesized to support Treg cell accumulation or function in tumors await detailed investigation.

Metabolic factors other than nutrients also contribute to heightened Treg cell accumulation and function in the tumor microenvironment. Hypoxia is common in some regions of tumor tissue due to lack of vascularity (215). HIF-1α is usually upregulated upon hypoxia, and is positively correlated with the malignancy of certain tumors (216). Previous studies have identified HIF-1α as a negative regulator of iTreg cell differentiation, while it promotes Th17 cell differentiation (78, 217). However, HIF-1α is required for optimal Treg cell suppressive activity *in vivo* (218). A study using mice with Treg cell-specific deletion of HIF-1α has revealed that, under hypoxia in a mouse model of glioma, HIF-1α directs glucose away from the mitochondria, leaving Treg cells dependent on fatty acids for mitochondrial metabolism (83). This mechanism provides an advantage for Treg cells to thrive in low-glucose tumor microenvironment. The tumor microenvironment also accumulates excess lactate produced by tumor cells due to their high rates of glycolysis (198). A major consequence of lactate secretion is microenvironmental acidification (198). Multiple studies have identified a deleterious role of lactate in inhibiting CD8^+^ T cell-mediated anti-tumor immunity, while neutralization of acidosis improves checkpoint blockade (219–221). In contrast, Treg cell suppressive function and proliferation are unaffected by the addition of lactate due to reduced glycolysis (17). Mechanistically, conventional effector T cells balance NAD^+^/NADH ratios through conversion of pyruvate into lactate to maintain glycolysis (17, 138), whereas Treg cells do not require high rates of glycolysis and can thus balance NAD^+^/NADH through oxidation of exogenous lactate (17). As a result, loss of glycolysis is detrimental to conventional effector T cells but not Treg cells (16), providing Treg cells with a metabolic advantage in response to low-glucose and high-lactate tumor microenvironment (17). Furthermore, oxidative stress is an additional metabolic feature in the tumor microenvironment (198). Treg cells are relatively more sensitive to oxidative stress than conventional T cells and undergo potent apoptosis in the tumor microenvironment (222). Intriguingly, apoptotic Treg cells have been revealed to mediate superior immunosuppression through converting a large amount of ATP to adenosine via CD39 and CD73 (222), suggesting that tumor-resident Treg cells sustain and amplify suppressive activity by inadvertent death via oxidative stress. These observations all underscore the importance of metabolic adaption of Treg cells in tumor microenvironment for their suppressive activity, although the precise metabolic status of tumor Treg cells needs to be determined, such as by single cell metabolomics (151) or computational-based inference of metabolic gene expression in scRNA-seq data (223).

## Concluding Remarks and Future Perspectives

Current studies illustrate that environmental and intracellular metabolic factors orchestrate Treg cell stability, plasticity, and tissue-specific heterogeneity in different contexts. How Treg cells metabolically adapt to different environmental contexts *in vivo* still remains underexplored. From the perspective of intricate molecular network, mTOR likely acts as a “metabolic hub” that senses and adjusts the cellular metabolic state, thereby balancing the catabolism and anabolism in Treg cells to define their functional and phenotypic state. Therefore, dissecting the upstream metabolic inputs to mTOR in Treg cells remains a key future direction and may uncover how environmental cues are transmitted to Treg cells for functional reprogramming. Apart from mTOR, the roles of other metabolic components, such as mitochondrial respiratory chain complexes, are being uncovered in Treg cells. It will be intriguing to determine whether the loss of various metabolic components within Treg cells causes distinct pathologies in mice. Utilizing CRISPR (Clustered Regularly Interspaced Short Palindromic Repeats) screening, novel metabolic regulators in Treg cell stability, plasticity and tissue-specific heterogeneity may be discovered, to further expand our view on Treg cell-specific metabolic regulation.

Unlike Treg cell instability, few studies have discussed the role of metabolism for Th-like Treg cell generation. The transcriptional landscape could be altered in Th-like Treg cells to allow the co-expression of Th transcription factors and inflammatory cytokines, along with Foxp3 expression, which may involve epigenetic regulation, such as histone acetylation. Metabolites such as acetyl-CoA can directly alter the activity of chromatin-modifying enzymes, including histone acetyl-transferases (HATs), histone methyl-transferases (HMTs) and sirtuins. It will be interesting to uncover the metabolic pathways and their key drivers that orchestrate the epigenetic landscape of the Th transcription factors and inflammatory cytokines in Th-like Treg cells. The development of ATAC-seq has allowed analysis of transcription factor occupancy in specific cell types, which provides a starting point for studying the interplay between metabolism and epigenetics in shaping Treg cell plasticity (224).

Technological advances, such as scRNA-seq, are likely to uncover how Treg cells metabolically adapt to different environments. For instance, a recent scRNA-seq analysis of colonic and skin Treg cells revealed that Treg cells undergo metabolic reprogramming as they migrate from lymphoid organ to non-lymphoid barrier organs (225). Specifically, Treg cells convert from expressing glycolysis- and migration-associated genes toward expressing genes associated with fatty-acid metabolism and cytokine production. Intriguingly, lymphoid organs are glucose-rich relative to non-lymphoid organs or tumor microenvironment (169), while non-lymphoid organs may express higher levels of fatty acids. These differences in local nutrient composition likely explain why Treg cells from adipose tissue and intestine express higher levels of the fatty acid-binding transporters than those in lymphoid tissues. The interplay between local nutrient composition and Treg cell stability, plasticity, and tissue-specific heterogeneity also likely accounts for the unique physiological functions of Treg cells in non-lymphoid organs, such as controlling adipose tissue homeostasis (226). As scRNA-seq, ATAC-seq, *in situ* imaging, and metabolic profiling have greatly expanded the possible view of cell-cell communication (227, 228), it may be possible to use these technologies to discover novel ligand-receptor pairs or signaling pathways responsible for Treg cell stability and plasticity in these sites. Recent work has also established that Treg cells from hypoxic tumors express higher fatty acid transporters and catabolize free fatty acids for immunosuppression (83). Thus, understanding the detailed mechanisms of metabolic cross-talk between Treg cells and tumor cells may uncover essential regulatory networks in tumorigenesis, tumor progression, and therapy resistance. Therefore, analysis of Treg cell-metabolic interactions may provide new opportunities to develop novel Treg cell-based immune-metabolic interventions for the treatment of inflammatory diseases and tumor progression.

## Author Contributions

All authors listed have made a substantial, direct and intellectual contribution to the work, and approved it for publication.

### Conflict of Interest

The authors declare that the research was conducted in the absence of any commercial or financial relationships that could be construed as a potential conflict of interest.
